# Supplementary material for: Challenges of Caregivers Regarding Homecare to Type 1 Diabetic Children in Vhembe District, South Africa: A Qualitative Study Report
Source: Nurs Rep. 2022 Nov 21;12(4):884–93. doi: 10.3390/nursrep12040085 (PMC9680399; doi:10.3390/nursrep12040085)
Supplement: Supplementary file 1 [file nursrep-12-00085-s001.zip › nursrep-2001601-supplementary.pdf]

Challenges of caregivers regarding homecare to Type-1 diabetic children in Vhembe District, South Africa

Consolidated criteria for reporting qualitative studies (COREQ): 32-item checklist

Developed from:

Tong A, Sainsbury P, Craig J. Consolidated criteria for reporting qualitative research (COREQ): a 32-item checklist for interviews and focus groups. *International Journal for Quality in Health Care*. 2007. Volume 19, Number 6: pp. 349 – 357

No. Item	Guide questions/description	Reported on Page #
**Domain 1: Research team and reflexivity**		
Personal Characteristics		
1. Inter viewer/facilitator	Which author/s conducted the inter view or focus group?<br>First author	Page 3
2. Credentials	What were the researcher's credentials?<br>E.g. Mcur	Page 3
3. Occupation	What was their occupation at the time of the study?<br>Registered nurse	Page 3
4. Gender	Was the researcher male or female?<br>Female	Page 3
5. Experience and training	What experience or training did the researcher have?<br>A registered nurse who is also pediatric trained	Page 3
Relationship with participants		
6. Relationship established	Was a relationship established prior to study commencement?<br>Yes	Page 3
7. Participant knowledge of the interviewer	What did the participants know about the researcher? e.g. personal goals, reasons for doing the research<br>Reasons for doing the research	Page 3

8. Interviewer characteristics	What characteristics were reported about the inter viewer/facilitator? e.g. Bias, assumptions, reasons and interests in the research topic	Page 3

**Domain 2: study design**		
Theoretical framework		
9. Methodological orientation and Theory	What methodological orientation was stated to underpin the study? e.g. grounded theory, discourse analysis, ethnography, phenomenology, content analysis<br>Qualitative study	Page 2
Participant selection		
10. Sampling	How were participants selected? e.g. purposive, convenience, consecutive, snowball<br>Purposively	Page 2
11. Method of approach	How were participants approached? e.g. face-to-face, telephone, mail, email<br>Face to face	Page 3
12. Sample size	How many participants were in the study?<br>15 participants	Page 2
13. Non-participation	How many people refused to participate or dropped out? Reasons?<br>None	
Setting		
14. Setting of data collection	Where was the data collected? e.g. home, clinic, workplace<br>Home	Page 2
15. Presence of non-participants	Was anyone else present besides the participants and researchers?<br>None	Page 3
16. Description of sample	What are the important characteristics of the sample? e.g. demographic data, date<br>Demographic data	Page 4
Data collection		
17. Interview guide	Were questions, prompts, guides provided by the authors? Was it pilot	Page 3

	tested?<br>Central questions was used followed by probing based on the participant's response. Pretest was also done	
18. Repeat interviews	Were repeat inter views carried out? If yes, how many?<br>No, repeat interviews were done. However, data saturated at 10th participant and the researcher continued till the 15th participant to ensure that nothing new is coming in	Page 2
19. Audio/visual recording	Did the research use audio or visual recording to collect the data?<br>Audio-tape	Page 3
20. Field notes	Were field notes made during and/or after the inter view or focus group?<br>Yes, during and after the interview	Page 3
21. Duration	What was the duration of the inter views or focus group?<br>30 to 45 minutes	Page 3
22. Data saturation	Was data saturation discussed?<br>Yes	Page 2
23. Transcripts returned	Were transcripts returned to participants for comment and/or correction?<br>Yes	Page 3
**Domain 3: analysis and findings**		
Data analysis		
24. Number of data coders	How many data coders coded the data?<br>one	Page 3
25. Description of the coding tree	Did authors provide a description of the coding tree?	
26. Derivation of themes	Were themes identified in advance or derived from the data?<br>Derived from the data	Page 3
27. Software	What software, if applicable, was used to manage the data?<br>Audio tape	Page 3
28. Participant checking	Did participants provide feedback on the	Page 3

	findings?<br>Yes	
Reporting		
29. Quotations presented	Were participant quotations presented to illustrate the themes/findings? Was each quotation identified? e.g. participant number<br>Yes	Page 5 to 7
30. Data and findings consistent	Was there consistency between the data presented and the findings?<br>Yes	Yes, there was.<br>Page 4 to 7
31. Clarity of major themes	Were major themes clearly presented in the findings?<br>Yes	Yes. they were.<br>From page 5 to 7
32. Clarity of minor themes	Is there a description of diverse cases or discussion of minor themes?<br>Yes	Discussion of major and minor themes<br>From page 7 to 9
